# Case Report: Novel *CSF1R* Variant in a Patient With Behavioral Variant Frontotemporal Dementia Syndrome With Prodromal Repetitive Scratching Behavior

**DOI:** 10.3389/fneur.2022.909944

**Published:** 2022-06-22

**Authors:** Adit Friedberg, Eliana Marisa Ramos, Zhongan Yang, Luke W. Bonham, Jennifer S. Yokoyama, Peter A. Ljubenkov, Kyan Younes, Daniel H. Geschwind, Bruce L. Miller

**Affiliations:** ^1^Department of Neurology, Memory and Aging Center, Weill Institute for Neurosciences, University of California, San Francisco, San Francisco, CA, United States; ^2^Global Brain Health Institute, University of California, San Francisco, San Francisco, CA, United States; ^3^Trinity College Dublin, Dublin, Ireland; ^4^Department of Neurology, David Geffen School of Medicine, University of California, Los Angeles, Los Angeles, CA, United States; ^5^Department of Psychiatry, David Geffen School of Medicine, University of California, Los Angeles, Los Angeles, CA, United States; ^6^Department of Radiology and Biomedical Imaging, University of California, San Francisco, San Francisco, CA, United States; ^7^Program in Neurogenetics, Department of Neurology, Center for Autism Research and Treatment, David Geffen School of Medicine, Semel Institute for Neuroscience and Human Behavior, University of California, Los Angeles, Los Angeles, CA, United States; ^8^Department of Human Genetics, David Geffen School of Medicine, University of California, Los Angeles, Los Angeles, CA, United States; ^9^Institute for Precision Health, University of California, Los Angeles, Los Angeles, CA, United States

**Keywords:** *CSF1R*-related leukoencephalopathy, frontotemporal dementia, repetitive behaviors, early symptoms, scratching

## Abstract

*CSF1R*-related leukoencephalopathy is an autosomal dominant neurodegenerative disease caused by mutations in the tyrosine kinase domain of the colony stimulating factor 1 receptor (CSF1R). Several studies have found that hematogenic stem cell transplantation is an effective disease modifying therapy however the literature regarding prodromal and early symptoms *CSF1R*-related leukoencephalopathy is limited. We describe a 63-year-old patient with 4 years of repetitive scratching and skin picking behavior followed by 10 years of progressive behavioral, cognitive, and motor decline in a pattern suggesting behavioral variant of frontotemporal dementia. Brain MRI demonstrated prominent frontal and parietal atrophy accompanied by underlying bilateral patchy white matter hyperintensities sparing the U fibers and cavum septum pellucidum. Whole-exome sequencing revealed a novel, predicted deleterious missense variant in a highly conserved amino acid in the tyrosine kinase domain of *CSF1R* (p.Gly872Arg). Given this evidence and the characteristic clinical and radiological findings this novel variant was classified as likely pathogenic according to the American College of Medical Genetics standard guidelines. Detailed description of the prodromal scratching and skin picking behavior and possible underlying mechanisms in this case furthers knowledge about early manifestations of *CSF1R*-related leukoencephalopathy with the hope that early detection and timely administration of disease modifying therapies becomes possible.

## Introduction

*CSF1R*-related leukoencephalopathy is an autosomal dominant neurodegenerative disease caused by mutations in the tyrosine kinase domain of the colony stimulating factor 1 receptor (*CSF1R*) (1). It accounts for ~10% of adult-onset leukodystrophies in European cohorts (2). Clinically, *CSF1R*-related leukoencephalopathy presents with variable combinations of progressive cognitive decline, neuropsychiatric symptoms, pyramidal weakness and spasticity, parkinsonism, gait impairment and seizures. Often the pattern of behavioral and emotional change is similar to behavioral variant frontotemporal dementia (bvFTD) with apathy as a particularly prominent feature (1, 3). Disease onset is typically in the fourth or fifth decade of life and progression is relentless with mean disease duration till death of 6.8 years (4). Consistent radiological features on brain magnetic resonance imaging (MRI) include white matter hyperintensities (WMH) primarily involving the frontal and parietal lobes, with sparing of the U-fibers. Usually, these WMH are bilateral, initially patchy evolving to confluency with restricted diffusion in many cases. Thinning of the corpus callosum is characteristic. Gray matter atrophy is evident over the frontal and parietal lobes. A high incidence of a cavum septum pellucidum and cavum vergae was observed in several case series (5–7). Brain CT demonstrates calcifications distributed in the frontal white matter adjacent to the anterior horns of lateral ventricles and in the parietal subcortical white matter.

The *CSF1R* gene encodes a transmembrane tyrosine kinase receptor expressed in the brain, predominantly in microglia. Binding of CSF1R to its ligands (CSF1 and interleukin-34) leads to initiation of signal transductions, which contribute to development, maintenance, and activation of microglia (8). In zebrafish models, haploinsufficiency in CSF1R leads to reduced microglial density and in postmortem human brain tissue from patients with *CSF1R*-related leukoencephalopathy widespread microglia depletion is also evident (9). Moreover, homozygous mutations in *CSF1R* result in a congenital absence of microglia, abnormal brain development, and pediatric-onset leukoencephalopathy (10). *CSF1R-*related leukoencephalopathy is increasingly conceptualized as a neurodegenerative disease that occurs in large part due to microglial dysfunction—a primary microgliopathy (11). There are now more than 70 different pathogenic variants in *CSF1R*, most of which occur in the tyrosine kinase domain resulting in disruption of protein function (1).

There is emerging evidence that hematopoietic stem cell transplantation (HSCT) is a disease modifying therapy for *CSF1R*-related leukoencephalopathy that stabilizes disease progression, highlighting the clinical importance of early detection of individuals with pathogenic *CSF1R* variants (12–14). Nevertheless, detailed description of early/prodromal behavioral changes in *CSF1R*-related leukoencephalopathy is limited.

In this case study we report a patient with a novel likely pathogenic variant in *CSF1R* who presented with repetitive scratching behavior 4 years prior to the onset of progressive relentless behavioral, cognitive and motor decline accompanied by radiologic features highly consistent with *CSF1R*-related encephalopathy. Our goals are to enhance the field's knowledge about early phenotypic and genotypic features of *CSF1R*-related leukoencephalopathy and to further elucidate the heterogenous genetic causes of bvFTD clinical syndrome. We also discuss possible mechanisms underlying the scratching behavior.

## Methods

Clinical and genetic evaluation was performed at the University of California, San Francisco, Memory and Aging Center as part of a prospective research study: “Frontotemporal Dementia: Genes, Images, and Emotions” (15). For patient anonymity some details have been modified.

Whole-brain structural MRI images were acquired using a 3T (Magnetom Prisma) scanner manufactured by Siemens implementing previously published acquisition protocols (16).

Whole-exome sequencing (WES) was conducted to screen known genes associated with dementia and leukoencephalopathies. Whole-exome regions were captured with the SeqCap EZ Human Exome Kit v3 and sequenced on an Illumina HiSeq4000 at the UCLA Neuroscience Genomics Core (semel.ucla.edu/ungc). Sequence reads were mapped to the GRCh37/hg19 reference genome and variants joint-called according to the Genome Analysis Toolkit best practices recommendations (17). Series of filtering steps were applied to prioritize variants, and candidate variants were confirmed by Sanger sequencing. The hexanucleotide repeat of *C9orf72* was screened using both fluorescent and repeat-primed PCR, as previously described (18).

## Results

### Case Description

L was a 63-year-old right-handed female who presented to evaluation in the memory clinic due to 10 years of progressive behavioral changes and cognitive decline. Her early developmental history was notable for mild reading and writing difficulties in elementary school. In her youth she suffered mild brain trauma, falling twice from her bike. With both events there was loss consciousness for a short duration and no residual symptoms. After high school L attended college, where she obtained a BA. She married in her twenties. Her baseline personality was described as delightful, kind and social. At the age of 45 she presented with an adult-onset epilepsy with generalized tonic-clonic seizures. The seizures were successfully controlled with valproic acid. At the age of 49 she developed a new behavior, repetitively scratching and picking the skin of her left torso and shoulder. She never explained to her family members the reason for the scratching and did not explicitly complain of pruritus. She did not report an increasing sense of tension prior to scratching or relief during or after the behavior. The scratching increased in frequency and severity. The behavior was not accompanied by any other perseverative, stereotyped or compulsive/ritualistic behaviors. She was examined by a dermatologist who found no causative skin lesion and defined her condition as “neurogenic itch.” Four years later, at the age of 53 years she became socially withdrawn and less communicative. Her husband lost substantial amounts of their money shopping but she took no action to protect herself. At the age of 55 years she could no longer manage her finances or run her own business. She was unable to operate her computer or other technological equipment. She asked her dentist to remove all her teeth and replace them with dentures, without expressing a reason for this request. At the age of 61 she started to purchase recklessly. She got into a physical fight with her husband and subsequently they divorced. L did not express concern about the divorce and seemed to show less empathy for others. She flew balloons in the neighborhood park for multiple hours a day. She also became hyperoral with preference to sweets. At the age of 63 when she presented to evaluation, the family noticed word finding difficulties. She gradually stopped talking but acknowledged people with eye contact and head movement. She undressed in front of her family and collected soda bottle caps. Her scratching behavior worsened, and she scratched her skin to the point of bleeding. Family history was taken for four generations and was unremarkable for either early onset dementia, psychiatric disease epilepsy or motor decline. Both of her parents lived into their eighties with no cognitive decline. Her paternal and maternal families originated from Western Europe. Neurological examination was remarkable for increased axial tone and rigidity and bradykinesia in her upper limbs more so on the right. In her mental status examination L seemed inattentive, abulic and stimulus bound. Her affect was flat. Verbal output was decreased with occasional Yes/No answers, and use of overlearned phrases such as “I don't think so.” She could not follow instructions required for praxis examination. Her Mini-Mental State Examination score was 6/30. General physical examination was unremarkable except for minor excoriations over her torso secondary to scratching.

At the time of presentation, 14 years after the emergence of the scratching behavior, her complete blood count, renal and liver function tests were within normal limits suggesting that systemic causes of pruritus such as a solid or hematologic malignancy, cholestasis or renal insufficiency were not likely. Blood electrolyte levels, thyroid functions, coagulation tests, HBA1C, B12 levels, methylmalonic acid levels and very-long-chain fatty acid (VLCFA), were also all within normal limits.

**Figure 1:**
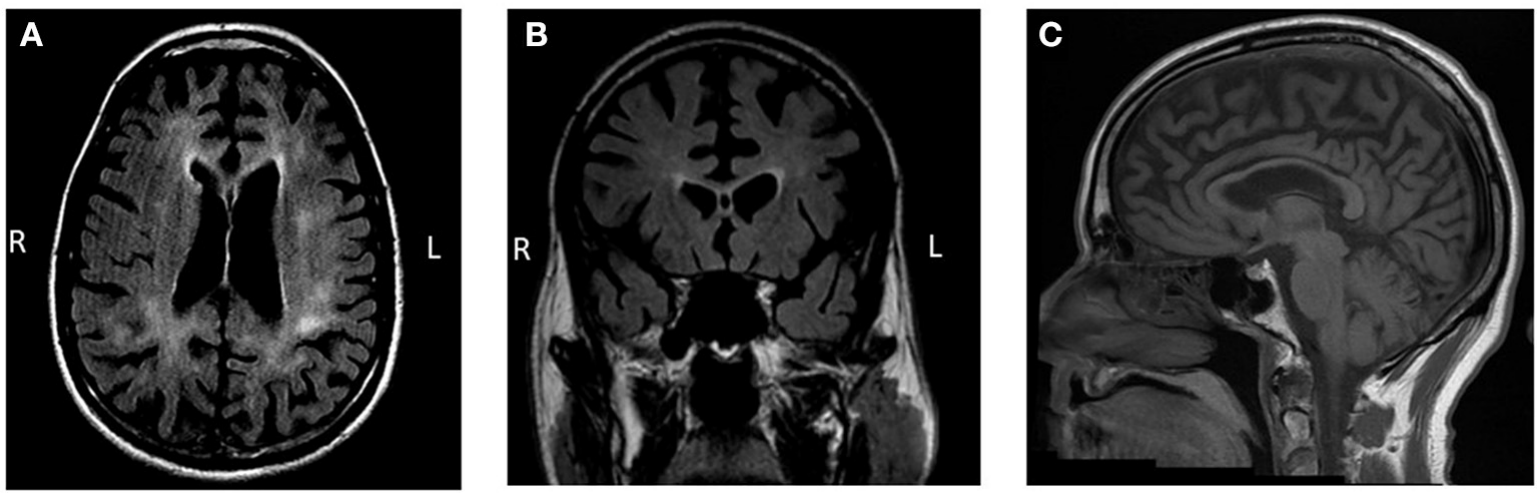
Brain MRI findings. **(A,B)** Bilateral patchy and predominantly frontoparietal white matter hyperintensities with accompanying marked gray matter atrophy are observed on T2-weighted fluid-attenuated inversion recovery (FLAIR) imaging. Cavum septum pellucidum is also evident. **(C)** Thinning of the corpus callosum is observed on a T1-weighted sequence.

Brain MRI demonstrated bilateral symmetrical global cortical atrophy (Figure 1). Atrophy was most pronounced in the frontal and parietal lobes, particularly in the medial frontal cortices. Profound thinning of the corpus callosum was evident. T2 FLAIR sequence showed bilateral patchy WMH involving frontal and parietal white matter, sparing the U fibers. Cavum septum pellucidum was noted. Diffusion weighted imaging did not show a pattern of restricted diffusion and T2^*^ sequences did not demonstrate signal abnormalities consistent with calcifications.

The clinical syndrome met research criteria for probable bvFTD (19). The combination of gray matter atrophy and WMH led the neurologists to consider underlying neuropathological etiologies associated with white matter signal changes such as corticobasal degeneration and frontotemporal lobar degeneration (FTLD) TDP type A. The pattern of white matter involvement in the setting of protracted clinical course was suggestive of leukodystrophy as a primary etiology, particularly *CSF1R*-related leukoencephalopathy considering the characteristic brain MRI findings (20) and seizures. To promote the diagnostic process WES was performed to analyze a large panel of genes associated with dementia and leukoencephalopathies.

**Figure 2:**
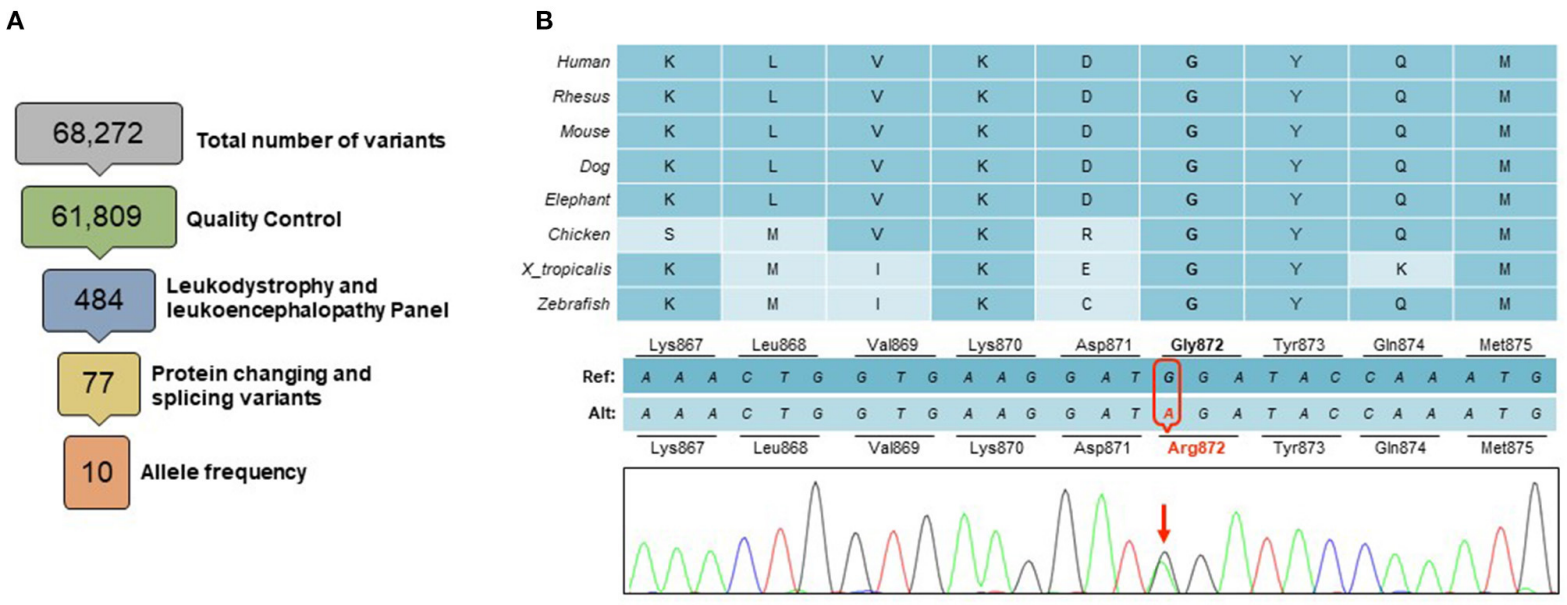
Genetic findings. **(A)** A total of 68,272 variants were identified within the targeted exome. Variants of low quality were removed through quality control (GQ <20, RD <10, AF <0.25). Among a panel of 165 genes known to be associated with leukodystrophies and leukoencephalopathies, a total of 484 variants were identified, of which 77 were protein changing or splicing variants. Only 10 of these had an allele frequency <0.10 in gnomAD database, including a novel heterozygous missense variant in the *CSF1R* gene **(B)** This change in a highly conserved amino acid in the CSF1R tyrosine domain (p.Gly872Arg), was predicted to be deleterious by multiple *in silico* tools.

WES revealed a novel heterozygous missense variant c.2614G>A (p.Gly872Arg) in the *CSF1R* gene (Figure 2). This variant, not found in the Genome Aggregation Database (gnomAD) (21), was consistently predicted as damaging by multiple *in silico* tools [PolyPhen-2 (http://genetics.bwh.harvard.edu/pph2), SIFT (http://sift.jcvi.org) and CADD (http://cadd.gs.washington.edu)]. It lies in the tyrosine kinase domain, similarly to many other *CSF1R*-related leukoencephalopathy causing variants, in a highly conserved amino acid. Segregation analysis of this variant was not feasible due to unreachable relatives of the proband. Given these evidences and the characteristic clinical and radiologic findings (22), this variant was classified as likely pathogenic according to the American College of Medical Genetics standard guidelines (23).

At the time L presented to our care, HSCT was not yet considered a therapeutic option and was not offered. L passed away due to her neurogenerative disease and was not enrolled into an autopsy program.

## Discussion

We present a patient with a progressive behavioral cognitive and motor syndrome compatible with bvFTD which was preceded by adult onset generalized epilepsy and 4 years of repetitive scratching and skin picking behavior. The pattern of white and gray matter changes, the profound thinning of the corpus callosum and the occurrence of septum pellucidum cavum were all suggestive of *CSF1R*-related leukoencephalopathy (22). Genetic analysis revealed a novel likely pathogenic missense variant in the *CSF1R* gene. Since both parents were not affected clinically this may represent a *de novo* genetic variant. Other possibilities include incomplete penetrance or genetic mosaicism as was shown in other sporadic cases (1, 24).

Repetitive scratching and skin picking behavior have not yet been described in the literature as an early or prodromal symptom of *CSF1R*-related leukoencephalopathy. Delineating and highlighting such symptoms may contribute to early detection of patients with pathogenic *CSF1R* variants, allowing timely treatment with new disease modifying therapies.

We suggest several possible mechanisms underlying the scratching behavior in the context *CSF1R*-related encephalopathy which are not mutually exclusive and may act synergistically. Scratching is considered a self-grooming behavior aimed to protect the organism of potential external threats and is observed in multiple species. In humans excessive scratching and skin picking, in a pattern like the behavior observed in this case, is observed in skin picking disorder (SPD) (25). A diffuse tensor imaging study in skin picking disorder patients demonstrated reduced white matter integrity in tracts in proximity of the bilateral anterior cingulate cortex (ACC) implicating the importance of the ACC in regulation of this behavior (26). Interestingly L presented with severe atrophy in the ACC bilaterally accompanied by adjacent white matter lesions (Figure 1). This pattern might indicate early regional involvement that accounted for the early onset of repetitive scratching and skin picking behavior. Moreover, a candidate animal model of skin picking disorder is the *Hoxb8* knockout mouse. Mice with mutations of the *Hoxb8* gene groom excessively, to the point of developing skin lesions (27). In this model *Hoxb8* is expressed in specific populations of microglia and neuronal cells (28). The *Hoxb8* gene is expressed in the orbital cortex, the anterior cingulate, the striatum, and the limbic system, structures implicated in repetitive and compulsive behaviors. Recent work in this mouse model suggested that the absence of proper maintenance of circuit modulation by *Hoxb8* expressing microglia results in the excessive grooming behavior (29). It is possible that early depletion of the *Hoxb8* expressing microglia population resulted in the predisposition to repetitive scratching behavior. Interestingly, in mouse models of FTD with progranulin deficiency, excessive grooming behaviors were present (30). This might imply converging neuroanatomical and/or underlying molecular mechanisms with scratching observed in *CSF1R*-related leukoencephalopathy. Lastly, in the previous decade CSF1R inhibitors were introduced in cancer treatment. Safety assessment of Emactuzumab, a monoclonal antibody that targets and inhibits CSF1R revealed that pruritus was a highly prevalent side effect affecting 56% of treated patients (31). Like other monoclonal antibodies Emactuzumab does not penetrate the blood brain barrier suggesting that CSF1R dysfunction in peripheral tissues might contribute to the scratching behavior.

### Limitations

Definite causal evidence with regards to the pathogenicity of this variant was not obtained due to the unavailability of neuropathological data and unreachable family members for segregation analysis. However, building on the knowledge accumulated from multiple studies about the underlying biology and the clinical and radiological presentation of *CSF1R*-related encephalopathy there is high likelihood that this variant is disease causing. Future work is needed to establish definite causal relationship between this novel genetic variant and *CSF1R*-related encephalopathy and to delineate the pattern of early behavioral manifestations in this condition.

## Data Availability Statement

The datasets presented in this article are not readily available because of ethical and privacy restrictions. Requests to access the datasets should be directed to the corresponding author.

## Ethics Statement

The studies involving human participants were reviewed and approved by University of California, San Francisco Human Research Protection Program Institutional Review Board. The patients/participants provided their written informed consent to participate in this study. Written informed consent was obtained from the individual(s) for the publication of any potentially identifiable images or data included in this article.

## Author Contributions

AF, ER, and BM conceptualized the study. AF conducted the literature review and wrote the manuscript. BM, KY, and PL examined the patient. ER, ZY, DG, LB, and JY were involved in genetic data generation, analysis, and interpretation. All authors reviewed and revised the final manuscript.

## Funding

This work was supported by NIH grants P01 AG019724 and P30 AG062422, by grant 2021-A-023-FEL from the Larry L. Hillblom Foundation (AF), by the Global Brain Health Institute, Alzheimer's Association, and Alzheimer's Society Pilot Awards for Global Brain Health Leaders: GBHI ALZ UK-21-721419 (AF) and NIH-NIA grant R01 AG062588 (JY). Samples from the National Cell Repository for Alzheimer's Disease (NCRAD), which receives government support under a cooperative agreement grant (U24 AG021886) awarded by the National Institute on Aging (NIA), were used in this study.

## Conflict of Interest

The authors declare that the research was conducted in the absence of any commercial or financial relationships that could be construed as a potential conflict of interest.

## Publisher's Note

All claims expressed in this article are solely those of the authors and do not necessarily represent those of their affiliated organizations, or those of the publisher, the editors and the reviewers. Any product that may be evaluated in this article, or claim that may be made by its manufacturer, is not guaranteed or endorsed by the publisher.
